# Single-bundle versus double-bundle autologous anterior cruciate ligament reconstruction: a meta-analysis of randomized controlled trials at 5-year minimum follow-up

**DOI:** 10.1186/s13018-018-0753-x

**Published:** 2018-03-10

**Authors:** Haitao Chen, Biao Chen, Kai Tie, Zhengdao Fu, Liaobin Chen

**Affiliations:** Department of Orthopedic Surgery, Zhongnan Hospital of Wuhan University, Wuhan, 430071 China

**Keywords:** Mid- to long-term outcome, Anterior cruciate ligament, Reconstruction, Single-bundle, Double-bundle, Meta-analysis

## Abstract

**Background:**

Both single-bundle (SB) and double-bundle (DB) techniques were widely used in anterior cruciate ligament (ACL) reconstruction recently. Nevertheless, up to now, no consensus has been reached on whether the DB technique was superior to the SB technique. Moreover, follow-up of the included studies in the published meta-analyses is mostly short term. Our study aims to compare the mid- to long-term outcome of SB and DB ACL reconstruction concerning knee stability, clinical function, graft failure rate, and osteoarthritis (OA) changes.

**Methods:**

This study followed the PRISMA (Preferred Reporting Items for Systematic Reviews and Meta-Analyses) guidelines. The PubMed, Embase, and the Cochrane Library were searched from inception to October 2017. The study included only a randomized controlled trial (RCT) that compared SB and DB ACL reconstruction and that had a minimum of 5-year follow-up. The Cochrane Collaboration’s risk of bias tool was used to assess the risk of bias for all included studies. Stata/SE 12.0 was used to perform a meta-analysis of the clinical outcome.

**Results:**

Five RCTs were included, with a total of 294 patients: 150 patients and 144 patients in the DB group and the SB group, respectively. Assessing knee stability, there was no statistical difference in side-to-side difference and negative rate of the pivot-shift test. Considering functional outcome, no significant difference was found in proportion with International Knee Documentation Committee (IKDC) grade A, IKDC score, Lysholm scores, and Tegner scores. As for graft failure rate and OA changes, no significant difference was found between the DB group and the SB group.

**Conclusion:**

The DB technique was not superior to the SB technique in autologous ACL reconstruction regarding knee stability, clinical function, graft failure rate, and OA changes with a mid- to long-term follow-up.

## Background

Anterior cruciate ligament (ACL) injuries destroy the normal kinematics of the knee and may be more likely to cause secondary injuries including meniscal injuries and knee osteoarthritis (OA) [1, 2]. ACL reconstruction is widely used to restore knee laxity, reestablish biomechanical homeostasis, and prevent the long-term joint degeneration [3–5]. In recent years, both single-bundle (SB) and double-bundle (DB) techniques were commonly used in ACL reconstruction [6, 7]. However, up to now, no consensus has been reached on whether the DB technique was superior to the SB technique.

It is well known that the ACL may be divided into two functional bundles, the anteromedial bundle (AMB) and the posterolateral bundle (PLB) [5, 8]. These two grafts cross each other inside the joint, acting separately at different knee angles. Theoretically, the AMB may prevent an anterior tibial translation at higher flexion angles, while the PLB may additionally restrain anterior tibial loads as well as a combined rotatory load at lower flexion angles [9]. Several biomechanical studies [10–14] reported that the DB technique could rebuild both the AMB and the PLB and thus might reproduce knee stability and kinematics closer to the native knee than the SB technique in ACL reconstruction. However, other biomechanical studies of Kondo et al. [15] as well as Lorbach et al. [16] showed that the DB reconstruction might not offer significant further advantages than the SB reconstruction. Previous clinical studies with short-term follow-ups also got an inconsistent result when comparing DB with SB ACL reconstruction. On the one hand, several studies [8, 17, 18] reported that the DB technique could achieve a superior result in both knee stability and clinical functions. Meanwhile, some literature [19–23] indicated that the DB technique could acquire better knee stability, but get comparable postoperative functions to the SB technique. On the other hand, several researchers [6, 22, 24–28] found that both knee stability and clinical functions had no significant difference between the two techniques in ACL reconstruction. Given the diverse results of previous studies, it is imperative to pool the data to compare the DB and SB techniques and thus provide a reference for ACL reconstruction.

A recent meta-analysis [29] of 26 randomized controlled trials (RCTs) showed that the DB technique could yield a better outcome in both functional outcome and stability of the knee than the SB technique in ACL reconstruction. In another meta-analysis, Li et al. [30] found that the DB ACL reconstruction had a better outcome in rotational stability, while there was no great difference in functional outcome between the DB and SB techniques. However, the above two studies [29, 30] failed to assess some outcome parameters, such as graft failure and OA changes, between the two techniques. Furthermore, the follow-up of most of the included studies in both meta-analyses is short term. It is well known that OA is a chronic progressive degenerative disease, which can be found through X-ray as early as 4 to 5 years postoperatively [31]. It is more persuasive and reliable to compare the DB and SB techniques in ACL reconstruction with a longer-term follow-up.

The purpose of this meta-analysis was to determine whether there is a significant difference in postoperative knee stability, clinical function, graft failure rate, and OA changes for DB versus SB technique in ACL reconstruction with a minimum of 5-year follow-up.

## Methods

### Literature search

This study was designed and conducted according to the PRISMA (Preferred Reporting Items for Systematic Reviews and Meta-Analyses) guidelines [32]. The PubMed, Embase, and the Cochrane Library were reviewed for all English language studies from inception to October 2017. Two independent reviewers (HTC and BC) searched each database using the following strategy: (“anterior cruciate ligament” OR ACL) AND (single-bundle OR “single bundle”) AND (double-bundle OR “double bundle”). A manual search for references of included articles was also conducted to ensure no eligible studies were missed.

### Inclusion criteria and exclusion criteria

Inclusion criteria were as follows: (1) subject—all patients who underwent arthroscopy-assisted ACL reconstruction, with no limitation to sex or race; (2) intervention method—comparison of clinical outcome between the SB and DB technique in autologous ACL reconstruction; (3) outcome parameters—side-to-side difference (SSD), pivot-shift tests, International Knee Documentation Committee (IKDC) grade A, IKDC scores, Lysholm scores, Tegner scores, graft failure, and OA changes; (4) study type—RCT.

The exclusion criteria were as follows: (1) non-prospective trials (e.g., retrospective studies, observational studies, case series, and reviews); (2) animal or cadaver studies; (3) comparisons that were not between SB and DB method in ACL reconstruction; (4) follow-up less than 5 years; and (5) allograft ACL reconstruction.

### Data extraction

Data from eligible studies were extracted independently by the two same reviewers according to predefined selected criteria, including article information (author and publication date), participant demographics, follow-up period, sample size, implant, femoral drilling technique, fixation type, and outcome parameter. The KT-1000 and KT-2000 arthrometers in the included studies were reported in the form of SSD. Disagreements on data extraction were resolved by discussion.

### Assessment of risk of bias

Two reviewers independently assessed the risk of bias for all included studies using the Cochrane Collaboration’s risk of bias tool, which contains six items as follows: random sequence generation (selection bias), allocation concealment (selection bias), blinding of participants and personnel (performance bias), incomplete outcome data (attrition bias), selective reporting (reporting bias), and other bias. Each of included studies was rated as having a low, unclear, or a high bias regarding the above items. Publication bias was not detected because of the limited number of included studies. Disagreements were resolved by discussion.

### Statistical analysis

The meta-analysis was conducted using Stata/SE version 12.0. When the outcome indicator was dichotomous outcomes, relative risk (RR) was calculated for effect size. For continuous outcomes, a weighted mean difference (WMD) was calculated when the same measurement criterion was used; otherwise, a standardized mean difference (SMD) was calculated both used 95% confidence intervals (CI). The intervening effect of an indicator was considered as zero difference if 95% CI for WMD or SMD contained 0 and 95% CI for RR contained 1. The statistical heterogeneity was tested with the chi-square test and *I*^2^. If heterogeneity was low (*P* > 0.1 or *I*^2^ ≤ 50%), a fixed effects model was used. If heterogeneity was significant (*P* < 0.1, *I*^2^ > 50%), sensitivity analysis, subgroup analyses, and meta-regression were conducted to find the source of the heterogeneity. If the heterogeneity could not be eliminated, a random effects model would be used when the result of meta-analysis had clinical homogeneity, or descriptive analysis would be used.

## Results

### Article selection results

**Fig. 1:**
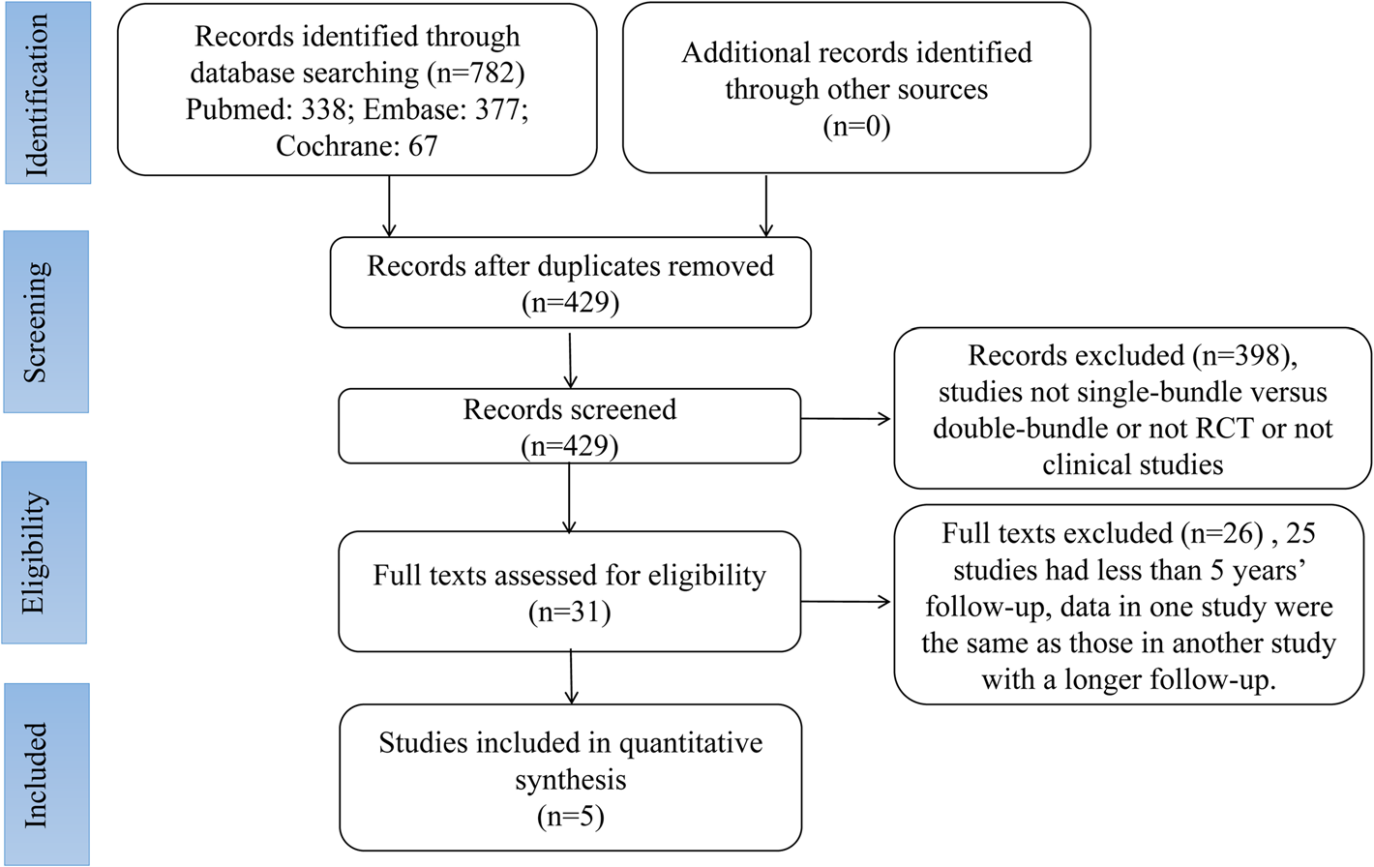
Flowchart of article selection process

Seven hundred eighty-two relevant articles were initially selected according to the search strategy. Three hundred fifty-three were excluded after checking for duplicates with the literature management software Endnote X7. Three hundred ninety-eight were excluded after reviewing the titles and the abstracts, 26 published articles were excluded by reviewing their full content as 25 studies had less than 5 years’ follow-up and data in one study were the same as those in another study with a longer follow-up. Finally, five articles [33–37] were included in the meta-analysis. A summary of the review process is presented in Fig. 1.

### Description of included studies

All five selected articles were written in English, which compared the clinical outcomes of the DB and SB techniques in ACL reconstruction. All follow-up periods in the included articles were ≥5 years. There was a total of 294 patients: 150 patients and 144 patients in the DB group and the SB group, respectively. All basic article information is reported in Table 1, and the mid- to long-term outcome measures of the two techniques are reported in Table 2.

**Table 1:** Characteristics of included studies

Study	Age, year Mean (SD)	Follow-up, year	*n* (the last follow-up)	Femoral drilling	Implant	Fixation		Outcome
						FS	TS	
Jarvela (2017) [33]	DB: 34 ± 10 SB: 30 ± 8	10	DB: 24 SB: 23	AM	HT (AU)	BIS	BIS	Lysholm score; IKDC score; IKDC grade A; pivot-shift test; KT-1000 (SSD); OA changes; revision surgery (graft failure)
Beyaz (2017) [34]	DB: 33.53 ± 5.47 SB: 31.06 ± 5.48	8	DB: 15 SB: 16	AM	HT (AU)	EB	BIS	Tegner activity scale; IKDC score; Lysholm score; OA changes; tunnel widening; Isokinetic muscle strength
Adravanti (2017) [35]	DB: 26.4 ± 8.5 SB: 28.3 ± 6.2	6	DB: 25 SB: 25	DB: TT (AMB), outside-in (PLB); SB:TT	HT (AU)	EB	BIS	Lysholm score; IKDC grade A; KT-2000 (SSD); OA changes; graft rerupture (graft failure)
Karikis (2016) [36]	DB: 33.53 ± 5.47 SB: 31.06 ± 5.48	5	DB: 46 SB: 41	AM	HT (AU)	MIS	BIS	Tegner level; Lysholm score; Single-legged hop test; KOOS Outcomes; KT-1000 (SSD); Lachman test; pivot-shift test; OA changes
Zaffagnini (2011) [37]	DB: 27 ± 9 SB: 26 ± 9.5	8	DB: 40 SB: 39	DB: medial portal; SB: AM	DB: HT (AU) SB: BPTB(AU)	IS	IS	IKDC grade A; pivot-shift test; Tegner level; KT-2000 (SSD)

*SD* standard deviation, *DB* double-bundle, *SB* single-bundle, *AM* anteromedial portal technique, *TT* transtibial technique, *HT* hamstring tendon, *BPTB* bone-patellar tendon-bone, *AU* autologous, *FS* femoral side, *TS* tibial side, *BIS* bioabsorbable screw, *MIS* metal interference screws, *IS* interference screws, *IKDC* International Knee Documentation Committee, *SSD* side-to-side difference, *KOOS* Knee injury and Osteoarthritis Outcome Score, *OA* osteoarthritis, *ROM* range of motion

**Table 2:** Mid- to long-term outcome measures of two techniques

Study	*N*	SSD^a^ (mm)		PS test (N/P)		IKDC A (Y/N)		IKDC scores^a^		Lysholm scores^a^		Tegner scores^a^		Graft failure (Y/N)		OA changes (Y/N)	
		DB	SB	DB	SB	DB	SB	DB	SB	DB	SB	DB	SB	DB	SB	DB	SB
Jarvela (2017) [33]	47	−0.1 ± 2	0.6 ± 1.9	23/1	23/0	19/5	18/5	9 ± 2	9 ± 2	94 ± 7	95 ± 7	–	–	1/23	7/16	12/12	8/15
Beyaz (2017) [34]	31	–	–	–	–	–	–	7.1 ± 0.91	7.1 ± 0.94	81.43 ± 6.45	81.94 ± 7.15	3.43 ± 1.34	3.47 ± 1.12	–	–	7/8	5/11
Adravanti (2017) [35]	50	1.4 ± 0.6	1.3 ± 0.8	–	–	13/12	13/12	–	–	96.4 ± 17.3	94.2 ± 15.3	–	–	1/24	0/25	3/22	2/23
Karikis (2016) [36]	87	2.2 ± 2.7	2.3 ± 2.7	32/4	38/7	–	–	–	–	90.1 ± 9.1	84.3 ± 21.2	5.7 ± 1.3	5.7 ± 1.5	–	–	8/30	11/34
Zaffagnini (2011) [37]	79	1.1 ± 1.9	0.4 ± 0.6	36/4	26/13	35/5	26/13	–	–	–	–	6 ± 2	4 ± 2	–	–	–	–

*SSD* side-to-side difference, *DB* double-bundle, *SB* single-bundle, *PS* pivot-shift, *N/P* negative/positive, *IKDC* International Knee Documentation Committee, *Y/N* yes/no, *OA* osteoarthritis

^a^The value is given as mean ± standard deviation

### Assessment of risk of bias

**Fig. 2:**
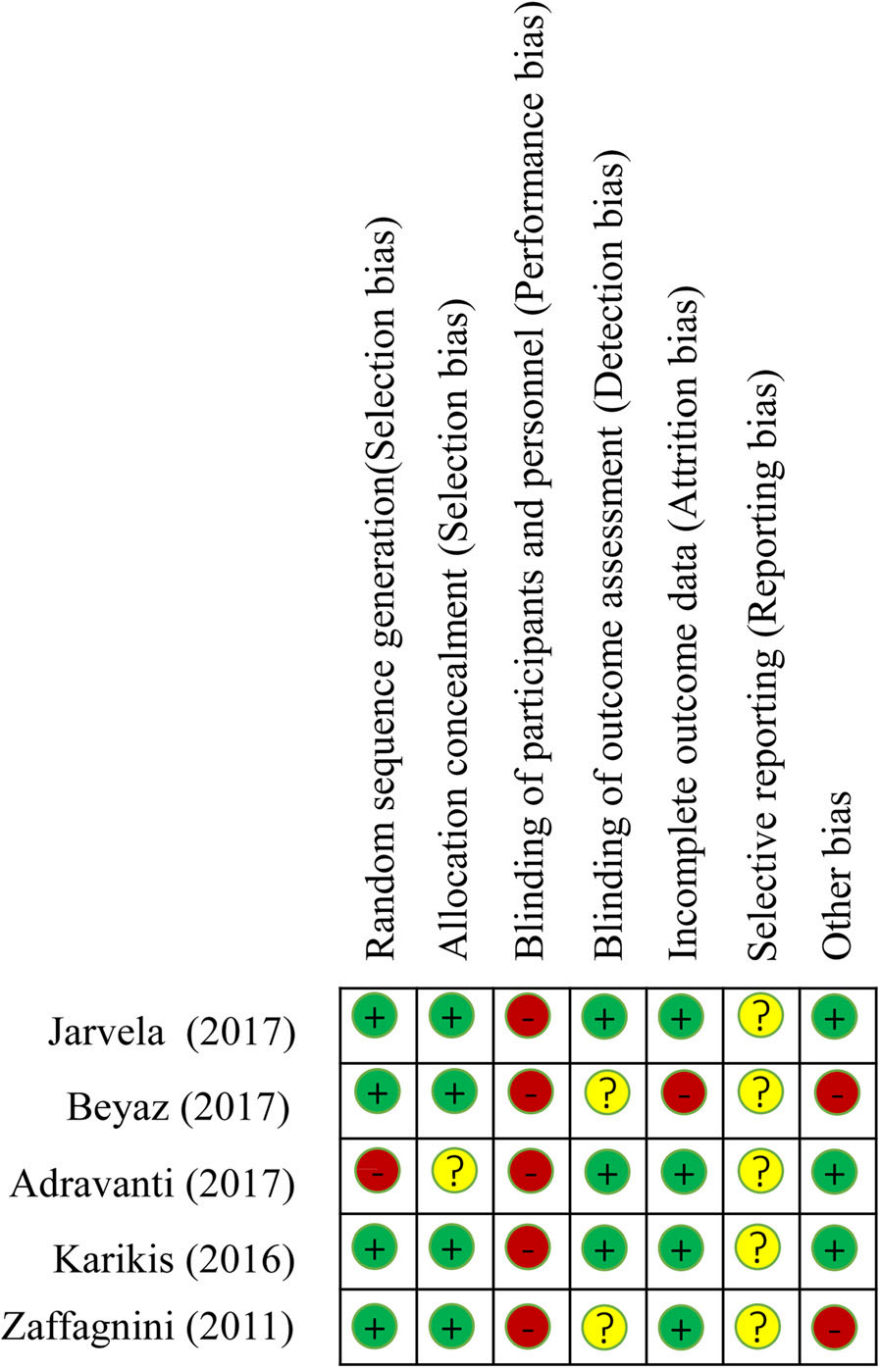
Assessment of risk of bias. +, low risk; −, high risk; ?, unknown risk

The results of the assessment of the risk of bias on included studies are summarized in Fig. 2. The study by Adravanti et al. [35] used a block randomization scheme to group the two treatments randomly, and thus this study was rated as having a high risk of selection bias, whereas the remaining studies were rated as having a low risk of selection bias. All included studies [33–37] failed to conduct the blinding therapists regarding DB or SB technique, and thus these were rated as having a high risk of performance bias. The studies by Beyaz et al. [34] and Zaffagnini et al. [37] did not describe the blinding of outcome assessment, and thus these were rated as an unknown risk for detection bias. One study [34] lost more than 20% of enrolled patients during follow-up and was regarded as having a high risk of attribution bias. All included studies [33–37] offered insufficient information to judge selective outcome reporting, and thus these were rated as having an unknown risk of reporting bias. One study [34] included only male patients and one study [37] used hamstring for DB technique ACL reconstruction and used bone-patellar tendon-bone for SB technique ACL reconstruction, and thus these were rated as having a high risk of potential other bias.

### SSD

**Fig. 3:**
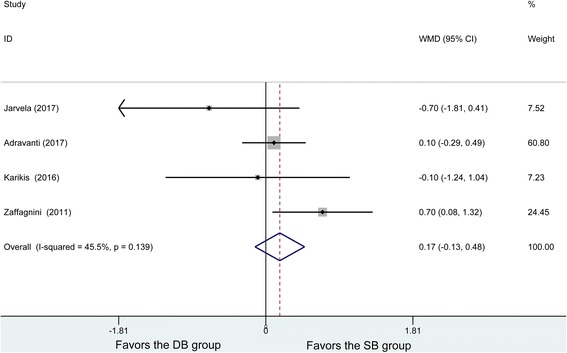
Forest plot of SSD. WMD, weighted mean difference

Four studies reported postoperative SSD, and no heterogeneity was found among the studies (*P* = 0.139, *I*^2^ = 45.5%). Using the fixed effects model, 135 patients in the DB and 128 patients in the SB group were analyzed with no significant difference in SSD (WMD = 0.17, 95% CI (− 0.13, 0.48), *P* = 0.27) (Fig. 3).

### Pivot-shift test

**Fig. 4:**
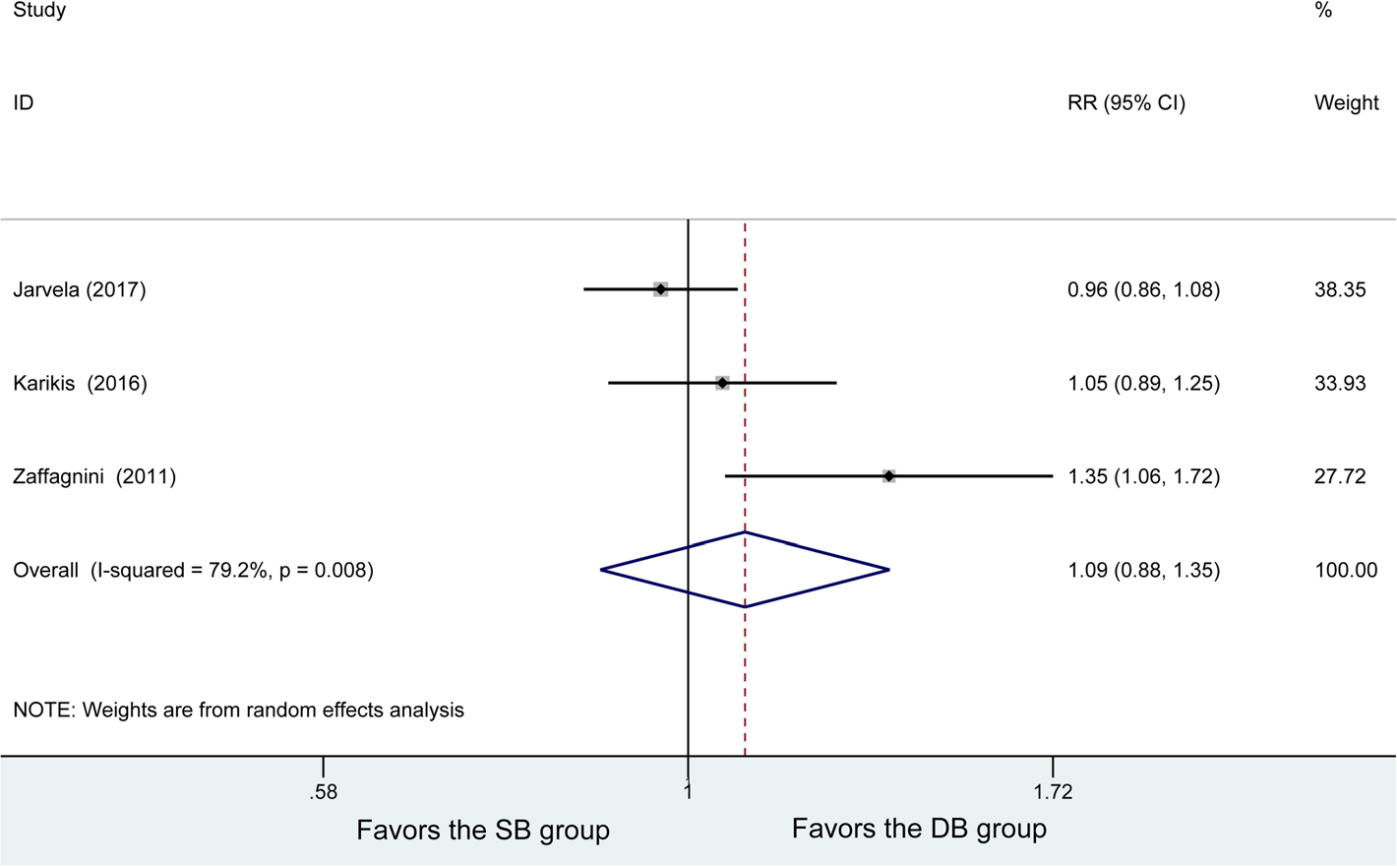
Forest plot of pivot-shift test

Postoperative pivot-shift tests were conducted in four studies. The analysis of negative pivot shift results showed some heterogeneity among the studies (*P* = 0.008, *I*^2^ = 79.2%). By using a random effects model, 100 patients in the DB group and 107 patients in the SB group were analyzed with no significant difference in postoperative negative pivot-shift (RR =1.09, 95% CI (0.88, 1.35), *P* = 0.441) (Fig. 4). Subsequently, to explore the potential source of heterogeneity, the pivot shift test was subjected to a sensitivity analysis by omitting one article at a time and calculating the pooled RRs for the remaining studies. It was found that there were no great changes in effect when any one study was excluded.

### IKDC grades

**Fig. 5:**
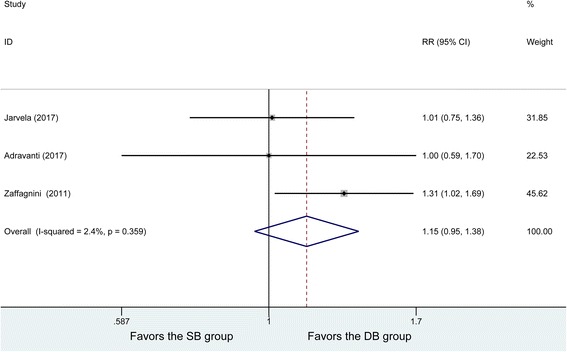
Forest plot of IKDC grades

Three studies included IKDC grades, and no heterogeneity was found among the studies (*P* = 0.359, *I*^2^ = 2.4%). Eighty-nine patients in the DB group and 87 patients in the SB group were analyzed using the fixed effects model, with no significant difference being found in proportion with IKDC grade A (RR = 1.15, 95% CI (0.95, 1.38), *P* = 0.156) (Fig. 5).

### IKDC scores

**Fig. 6:**
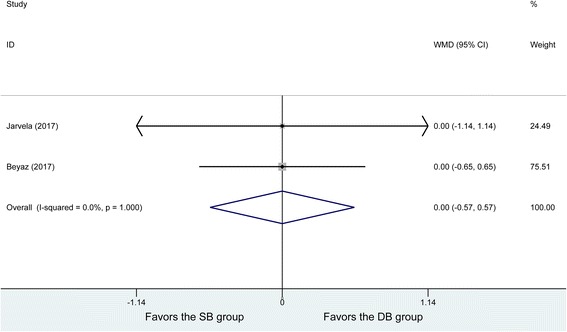
Forest plot of IKDC scores

Two studies demonstrated postoperative IKDC scores, with no heterogeneity being found between the studies (*P* = 1, *I*^2^ = 0%). Thirty-nine patients in the DB group and 39 patients in the SB group were analyzed using the fixed effects model, and no significant difference was found in the postoperative IKDC scores (WMD = 0, 95% CI (− 0.57, 0.57), *P* = 1) (Fig. 6).

### Lysholm scores

**Fig. 7:**
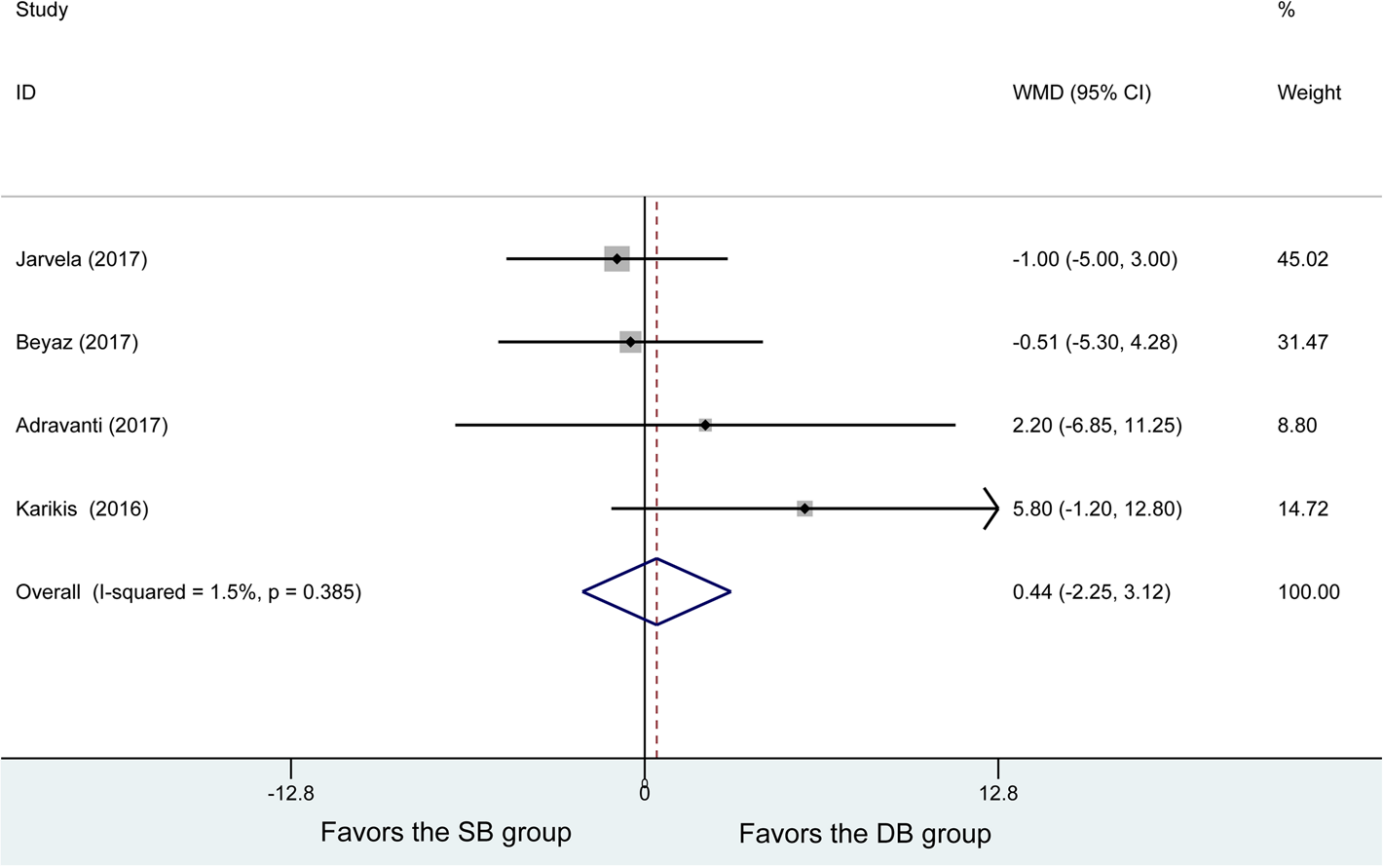
Forest plot of Lysholm scores

Four studies reported postoperative Lysholm scores, with no heterogeneity being found among the studies (*P* = 0.385, *I*^2^ = 1.5%). One hundred ten patients in the DB and 105 patients in the SB group were analyzed using the fixed effects model, and no significant difference was found in the postoperative Lysholm scores (WMD = 0.44, 95% CI (− 2.25, 3.12), *P* = 0.75) (Fig. 7).

### Tegner scores

**Fig. 8:**
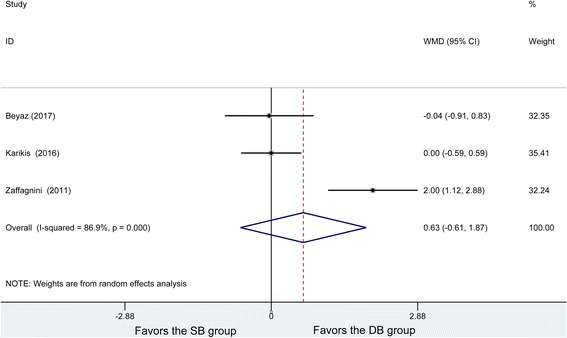
Forest plot of Tegner scores

Three studies reported postoperative Tegner scores, and obvious heterogeneity was found among these studies (*P* = 0, *I*^2^ = 86.9%). The random effects model was used to analyze 101 patients in the DB group and 96 patients in the SB group, showing no significant difference in postoperative Tegner scores (WMD = 0.63, 95% CI (− 0.61, 1.87), *P* = 0.317) (Fig. 8). Subsequently, to explore the potential source of heterogeneity, the Tegner scores were subjected to a sensitivity analysis by omitting one article at a time and calculating the pooled WMDs for the remaining studies. It was found that there were no great changes in effect when any one study was excluded.

### Graft failure

**Fig. 9:**
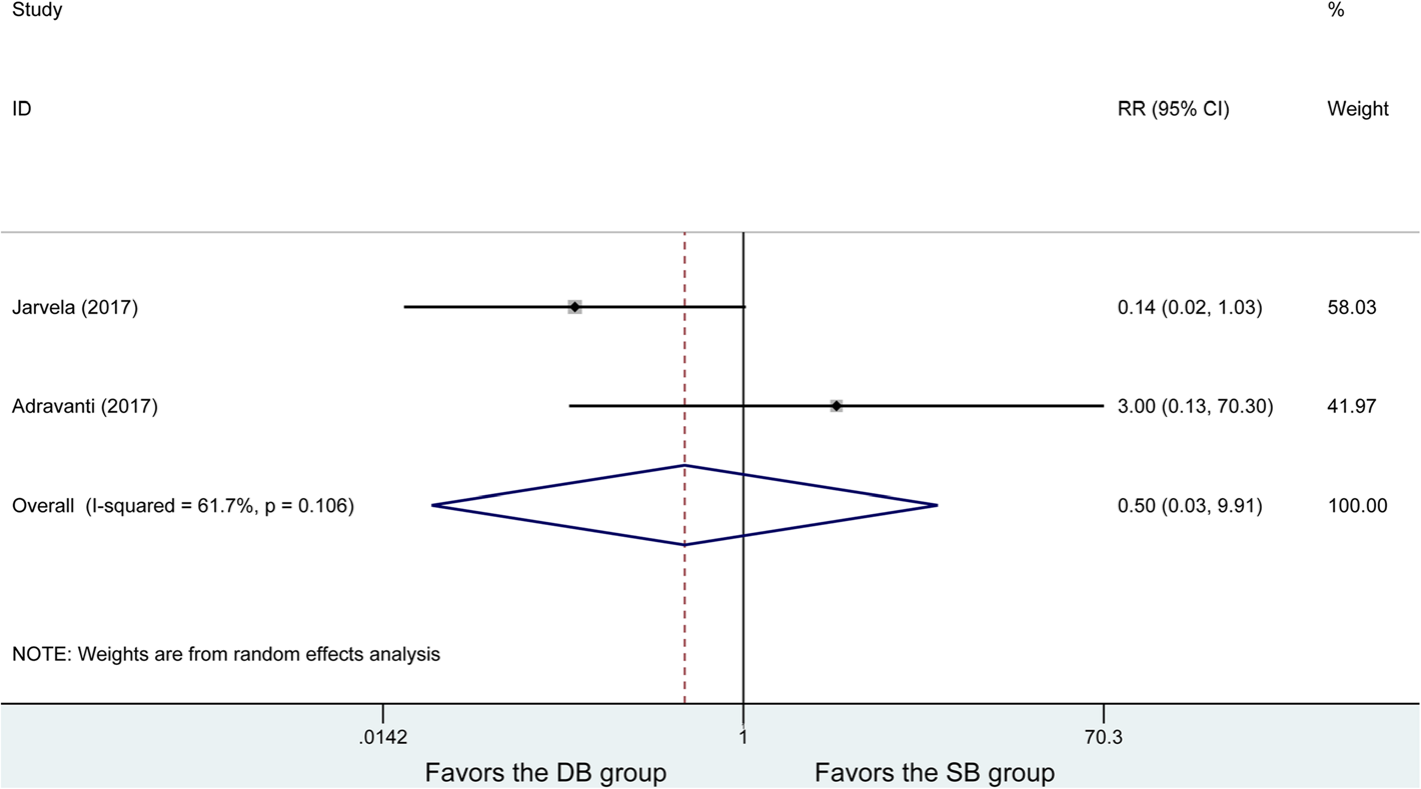
Forest plot of graft failures

Graft failure was conducted in two studies, with obvious heterogeneity between the studies (*P* = 0.106, *I*^2^ = 61.7%). The random effects model was used to analyze 49 patients in the DB group and 48 patients in the SB group, showing no significant difference in postoperative graft failure rate (RR =0.5, 95% CI (0.05, 9.91), *P* = 0.649) (Fig. 9).

### OA

**Fig. 10:**
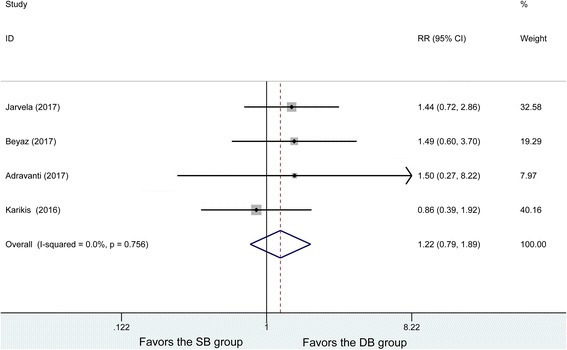
Forest plot of OA

Four studies included OA, and no heterogeneity was found between the studies (*P* = 0.756, *I*^2^ = 0%). The 102 patients in the DB group and 109 patients in the SB group were analyzed using the fixed effects model, with no significant difference being found in OA changes (RR = 1.22, 95% CI (0.79, 1.89), *P* = 0.37) (Fig. 10).

## Discussion

This meta-analysis was performed to compare the mid- to long-term outcome of the DB and SB techniques in autologous ACL reconstruction. The analysis included five RCTs involving 294 patients with at least 5 years of follow-up. The results revealed that there was no significant difference in knee stability, clinical function, graft rupture, and OA changes between the DB and SB techniques in autologous ACL reconstruction.

It is important to restore both the anterior and rotational stability in ACL reconstruction, which may be correlated with risk of meniscus and cartilage injury, as well as graft rupture and OA changes [2]. In our current study, all four included studies [33, 35–37] found that no statistical difference was found in anterior stability regarding KT-1000 or KT-2000 measurements. It was in line with the previous studies [6, 24, 31]. The authors of these studies reported that both the DB and SB techniques could closely imitate the AMB in ACL reconstruction and thus acquire comparable anterior stability. As for the rotational stability, two included studies [33, 36] found no great difference between the DB and SB techniques in ACL reconstruction, whereas one included study [37] showed that the DB technique could yield superior result than the SB technique. Theoretically, the DB technique also reconstructed the PLB, which functioned at extension and contributed more to rotational stability. However, our meta-analysis indicated that there was no significant difference between the DB and SB techniques in rotational stability. Hemmerich et al. [38] thought that the ACL could restrict the rotation of the knee, but its contribution to joint stability was limited under isolated torsional load. Furthermore, other authors [39, 40] suggested that peripheral knee structures, such as collateral ligaments and the musculature that crosses the knee joint, along with ACL played an important role in rotational stability.

In our study, clinical function showed no statistical difference between the DB and SB techniques in autologous ACL reconstruction. Four included articles [33–36] found that the DB technique in ACL reconstruction was not superior to the SB technique regarding the function parameters, including the Lysholm scores, the proportion with IKDC grade A, IKDC scores, and the Tegner scores. One included study [37] show that the DB technique could yield better functions than the SB technique in ACL reconstruction. In this study, the DB ACL reconstruction used an anatomical technique, while the SB ACL reconstruction used a non-anatomical technique. Furthermore, the grafts were also different in ACL reconstruction. That is, autologous hamstring graft was used in the DB technique, whereas autologous bone-patellar tendon-bone graft was used in the SB technique. This subtle difference of femoral drilling techniques and types of graft might influence the assessment of functional outcome and thus affect the accuracy of the result. Meanwhile, it might account for the difference between the one and the other four included studies.

Graft failure increases the future economic burden and individual suffering. Unfortunately, 0.7–20% of patients experience recurrent instability due to graft failure [41, 42]. In our meta-analysis, graft failure was referred to in two included studies. One study [33] reported that the DB ACL reconstruction resulted in significantly fewer graft failures than the SB ACL reconstruction. In this study, Jarvela et al. thought that the DB graft was stronger and might mimic the normal ACL anatomy more closely than the SB graft, and thus the DB technique was less likely to cause graft failure. However, the other study [35] found no great difference between the two techniques. In general, it is noteworthy that the cause of graft failure after ACL reconstruction is not solely influenced by the DB and SB techniques but also largely influenced by other risk factors, such as new knee trauma, infection of implanted graft, returning too soon to pivoting sports, and radical rehabilitation program [33]. In our current study, the DB technique had no obvious advantage in graft failure than the SB technique.

OA changes were also discussed in our meta-analysis. Three included studies found no great difference between the DB and SB techniques, whereas one included study showed more OA changes in the SB ACL reconstruction. The DB technique, in theory, could better delay the degeneration of knee than the SB technique in ACL reconstruction. Tajima et al. [43] and Morimoto et al. [14], for example, thought that SB ACL reconstruction might result in a significantly smaller patellofemoral and tibiofemoral contact area and higher pressures and thus had more OA changes. However, Jarvela et al. [33] found that the delay from the primary injury to ACL reconstruction affected OA changes. Also, some studies [31, 35, 44] reported the concomitant injury, such as meniscal or another ligament tear, as well influenced OA changes. In our study, the DB technique had no great difference with the SB technique in OA changes. Tunnel widening may lead to the inability of the implanted graft, long-term joint laxity, and difficulty in revision surgery [34, 45]. However, only one included RCT touched upon tunnel widening, and thus it was not suitable for conducting a meta-analysis. More prospective long-term RCTs are needed for future meta-analysis as for tunnel widening.

The advantage of this meta-analysis is that all the included studies were prospective RCTs with a minimal 5-year follow-up. Graft failure and OA changes usually needed to be assessed with a longer-term follow-up. Furthermore, a mid- and long-term result could offer a more persuasive and believable assessment of the stability and functional outcome and thus provide a reference for the choice of techniques in ACL reconstruction.

The limitations of this study were as follows: (1) The whole sample size was not large, and the outcome indicator was not unified, which may have influenced the outcome. (2) The femoral drilling technique and fixation technique in the studies were not all the same, which may not have been sufficiently homogeneous to evaluate the differences between the DB and SB techniques. (3) Several indicators, including KOOS outcomes, Lachman test, and tunnel enlargement were referred to in only one of the included study and could not be used as outcome parameters in the present study.

## Conclusion

The DB technique is not superior to the SB technique in autologous ACL reconstruction regarding knee stability, clinical function, graft failure rate, and OA changes with a mid- to long-term follow-up.

## Abbreviations

ACL: Anterior cruciate ligament
AMB: Anteromedial bundle
CI: Confidence intervals
DB: Double-bundle
IKDC: International Knee Documentation Committee
OA: Osteoarthritis
PLB: Posterolateral bundle
PRISMA: Preferred Reporting Items for Systematic Reviews and Meta-Analyses
RCTs: Randomized controlled trials
RR: Relative risk
SB: Single-bundle
SMD: Standardized mean difference
SSD: Side-to-side difference
WMD: Weighted mean difference
